# Skill Enactment and Knowledge Acquisition in Digital Cognitive Behavioral Therapy for Depression and Anxiety: Systematic Review of Randomized Controlled Trials

**DOI:** 10.2196/44673

**Published:** 2023-05-31

**Authors:** Hayley M Jackson, Alison L Calear, Philip J Batterham, Jeneva L Ohan, Glenda M Farmer, Louise M Farrer

**Affiliations:** 1 Centre for Mental Health Research National Centre for Epidemiology and Population Health The Australian National University Acton ACT Australia; 2 School of Psychological Science University of Western Australia Perth Australia

**Keywords:** cognitive behavioral therapy, technology, engagement, skill enactment, knowledge acquisition, depression, anxiety, adults, young people, systematic review, mobile phone

## Abstract

**Background:**

Digital cognitive behavioral therapy (CBT) interventions can effectively prevent and treat depression and anxiety, but engagement with these programs is often low. Although extensive research has evaluated program use as a proxy for engagement, the extent to which users acquire knowledge and enact skills from these programs has been largely overlooked.

**Objective:**

This study aimed to investigate how skill enactment and knowledge acquisition have been measured, evaluate postintervention changes in skill enactment and knowledge acquisition, examine whether mental health outcomes are associated with skill enactment or knowledge acquisition, and evaluate predictors of skill enactment and knowledge acquisition.

**Methods:**

PubMed, PsycINFO, and Cochrane CENTRAL were searched for randomized controlled trials (RCTs) published between January 2000 and July 2022. We included RCTs comparing digital CBT with any comparison group in adolescents or adults (aged ≥12 years) for anxiety or depression. Eligible studies reported quantitative measures of skill enactment or knowledge acquisition. The methodological quality of the studies was assessed using the Joanna Briggs Institute Critical Appraisal Checklist for RCTs. Narrative synthesis was used to address the review questions.

**Results:**

In total, 43 papers were included, of which 29 (67%) reported a skill enactment measure and 15 (35%) reported a knowledge acquisition measure. Skill enactment was typically operationalized as the frequency of enacting skills using the completion of in-program activities (ie, formal skill enactment; 13/29, 45%) and intervention-specific (9/29, 31%) or standardized (8/29, 28%) questionnaires. Knowledge measures included tests of CBT knowledge (6/15, 40%) or mental health literacy (5/15, 33%) and self-report questionnaires (6/15, 40%). In total, 17 studies evaluated postintervention changes in skill enactment or knowledge acquisition, and findings were mostly significant for skill enactment (6/8, 75% of the studies), CBT knowledge (6/6, 100%), and mental health literacy (4/5, 80%). Of the 12 studies that evaluated the association between skill enactment and postintervention mental health outcomes, most reported ≥1 significant positive finding on standardized questionnaires (4/4, 100%), formal skill enactment indicators (5/7, 71%), or intervention-specific questionnaires (1/1, 100%). None of the 4 studies that evaluated the association between knowledge acquisition and primary mental health outcomes reported significant results. A total of 13 studies investigated predictors of skill enactment; only type of guidance and improvements in psychological variables were associated with increased skill enactment in ≥2 analyses. Predictors of knowledge acquisition were evaluated in 2 studies.

**Conclusions:**

Digital CBT for depression and anxiety can improve skill enactment and knowledge acquisition. However, only skill enactment appears to be associated with mental health outcomes, which may depend on the type of measure examined. Additional research is needed to understand what types and levels of skill enactment and knowledge acquisition are most relevant for outcomes and identify predictors of these constructs.

**Trial Registration:**

PROSPERO CRD42021275270; https://www.crd.york.ac.uk/prospero/display_record.php?RecordID=275270

## Introduction

### Background

Depression and anxiety are common mental disorders [1,2], with estimated 1-year global prevalence rates ranging from 5% to 7% for major depressive disorder and 5% to 10% for anxiety disorders [3-5]. Despite the high level of burden associated with these disorders, many people do not seek professional help [6,7]. The provision of evidence-based digital mental health interventions (DMHIs) such as cognitive behavioral therapy (CBT) is proposed as a strategy to address this unmet need. Relative to in-person interventions, DMHIs can improve the reach and accessibility of care, are often available for free or at a lower cost, and may overcome several attitudinal barriers (eg, concerns about stigma and a preference for self-management) [8,9]. These programs also offer scalable solutions when traditional mental health services are displaced or demand for support increases, such as during public health crises [10]. Digital CBT programs deliver cognitive and behavioral skills and information via websites, smartphone apps, virtual reality, video games, or conversational agents [11]. There is considerable evidence showing that guided and unguided digital CBT can be effective in preventing and treating symptoms of depression and anxiety in adults [12-14] and young people [15,16].

Despite the promise of DMHIs, limited or low engagement with these programs is common. A systematic review of self-guided interventions for depression (most of which were based on CBT) found that >80% of research trial participants failed to complete all intervention modules, and only approximately 40% completed half of all modules [17]. Engagement with these programs in community settings is often much lower [18,19] as research trials typically recruit motivated participants, include human contact, and use explicit assessment procedures. Developing a better understanding of program engagement represents an important goal for research on DMHIs as there is meta-analytic evidence indicating that greater engagement is associated with better postintervention mental health outcomes [20,21]. However, notably, there is also limited evidence regarding what level of engagement, or dosage, is sufficient to achieve the intended outcomes [22], and some studies have demonstrated that outcomes are not always linearly related to engagement [23]. Nevertheless, research aimed at identifying the characteristics of individuals and interventions that influence engagement may enable the targeted development of strategies to facilitate engagement.

However, challenges remain regarding how to define and measure engagement with DMHIs [22]. Beintner et al [24] found that a range of indicators can be used to quantify engagement or adherence, the most common of which include indicators of module progression (eg, the percentage of participants completing a full intervention or completion of a minimum number of modules), log-ins, and time spent using the program. These metrics capture the extent to which individuals are exposed to intervention materials or progress through an intervention but may not elucidate engagement with other relevant behaviors that may also be important for mental health outcomes (eg, enactment of skills or accessing crisis support) [25]. Accordingly, there is current evidence suggesting that, although improvements in mental health outcomes are modestly associated with the number of modules accessed, they may not be associated with other use indicators [21,22]. The limitations of existing program use measures are further reinforced by observations that some individuals do not need to complete a full intervention to achieve symptom benefits (ie, e-attainers or early completers) [26,27].

### Skill Enactment and Knowledge Acquisition

Recognizing the need to improve how engagement with DMHIs is measured and reported, recent recommendations have proposed the use of multiple approaches [24,25,28], with a particular focus on measures that may be more indicative of effective engagement (ie, engagement necessary to produce outcomes) [29]. Evaluating whether individuals implement the strategies and techniques they have learned from a DMHI in their daily lives (hereafter referred to as skill enactment) may be especially important [30]. For example, Donkin et al [31] recommended using measures to assess the real-world implementation of skills taught during web-based interventions and suggested that this may be central to understanding the impact of engagement on mental health outcomes. Similar recommendations were provided in at least 3 subsequent reviews [24,25,28].

The importance of skill enactment for positive outcomes has also been instantiated in several frameworks of engagement with face-to-face and digital health interventions [32-34], which broadly correspond to some cognitive behavioral theories of the mechanisms of action in CBT [35,36]. These frameworks generally characterize enacting skills as an important causal mechanism by which interventions produce outcomes and, in particular, that program use (or attendance at in-person sessions) results in improvements in relevant health or mental health outcomes when an individual implements the strategies taught in a program effectively [32-34]. The literature on CBT skill use and emotion regulation extends this idea, suggesting that improvements in outcomes may occur through improvement in one of several skill enactment domains, including the frequency (ie, how often a person uses skills, regardless of the skill type), quantity (ie, the range of strategies used), or quality (ie, how well a person enacts skills in line with how they were taught) of skill enactment [35,37]. A further distinction is made between formal skill enactment (eg, completing a thought record) and informal skill enactment (eg, briefly reviewing the evidence against a negative automatic thought) [35]. However, questions remain regarding how program participation can produce changes in skill enactment and what factors might affect this process.

A factor that has been suggested to be important for both skill enactment and mental health outcomes is the acquisition of knowledge about mental health and awareness of strategies to address symptoms (hereafter referred to as knowledge acquisition) [33,38]. In comprehensive CBT interventions for depression and anxiety, psychoeducational components in the form of text, video, or audio tutorials are usually introduced early in the intervention process to improve knowledge and understanding of the signs and symptoms of depressive or anxiety disorders. Psychoeducation may help improve an individual’s ability to recognize and appropriately manage symptoms of depression or anxiety and has been shown to result in small improvements in symptoms even in the absence of other strategies [39]. In addition, information and instructions on the rationale for CBT and strategies for actively managing symptoms (eg, cognitive restructuring and behavioral activation) are provided throughout an intervention. If an individual does not acquire or understand the information related to these strategies, effective implementation and, thus, improvement in mental health outcomes are unlikely [38,40].

Although there is robust evidence for the effectiveness of digital CBT and the logical and theoretical importance of knowledge acquisition and skill enactment in achieving these outcomes, little is known about whether users acquire knowledge or enact skills from these interventions. Consequently, it is unclear whether skill enactment and knowledge acquisition are important for mental health outcomes or which factors influence skill enactment and knowledge acquisition. Although several reviews have investigated the predictors and outcomes of engagement or adherence [17,21,22,41,42], these studies have focused predominantly on indicators of program use (eg, module completion). Some reviews have also been conducted to evaluate the effectiveness of digital interventions in improving mental health literacy and its impact on mental health–related outcomes [43-45]. However, these previous reviews have not examined specialized knowledge acquired during participation in digital CBT interventions. Moreover, it is also important to examine how skill enactment and knowledge acquisition have been measured and reported in the literature to examine the extent to which measurement approaches align with theoretical conceptualizations of these concepts and to identify recommendations for future research on engagement with DMHIs.

### This Review

This systematic review builds on previous reviews by investigating skill enactment as a component of engagement with digital CBT interventions for depression and anxiety. Although we focused on skill enactment, the review adhered to a broader definition of engagement that includes initial uptake, ongoing use of a program, and enactment of skills in everyday life [33]. Given that knowledge acquisition is proposed to be essential for the enactment of skills and may itself be an important determinant of outcomes, we also reported data on knowledge acquisition as a secondary objective. Our specific review questions were as follows: (1) What methods have been used to measure skill enactment and knowledge acquisition in randomized controlled trials (RCTs) of digital CBT interventions for depression and anxiety? (2) Are digital CBT programs effective in improving skill enactment and knowledge acquisition? (3) Is there an association between mental health outcomes and skill enactment or knowledge acquisition? and (4) What predictors of skill enactment and knowledge acquisition have been identified?

## Methods

### Protocol

This review was reported according to the PRISMA (Preferred Reporting Items for Systematic Reviews and Meta-Analyses) guidelines [46] (refer to Multimedia Appendix 1 [46] for a PRISMA checklist) and was prospectively registered in PROSPERO (registration CRD42021275270). The review team originally intended to investigate skill enactment and knowledge acquisition in technology-based skills training interventions for depression and anxiety from any therapeutic orientation. However, we subsequently limited the scope of the review to digital CBT programs to facilitate comparisons across studies and because of the established evidence base for CBT in the treatment of depression and anxiety [16,47]. We added a criterion to exclude studies targeting people with physical illnesses given the potential for these programs to target specific types of knowledge and skills associated with the target condition. These decisions were made before the completion of screening and were added to the registered protocol as an amendment on March 24, 2022. No other substantive changes were made to the original protocol.

### Search Strategy

We searched the Cochrane CENTRAL, PubMed, and PsycINFO databases for peer-reviewed articles published between January 1, 2000, and July 26, 2022. The start date was selected to coincide with the publication of RCTs related to the first digital interventions for depression and anxiety [48,49]. The search strategy used combinations of relevant Medical Subject Heading terms and keywords for “technology-based intervention,” “randomized controlled trial,” and “depression” or “anxiety.” The full search strategy for each database is provided in Multimedia Appendix 2. Searches were rerun before final analyses. In addition, we supplemented the electronic search with forward citation searching of published self-report measures of CBT skill use to identify studies that may have been missed in the text-based searches. Citation searching is an effective way to retrieve studies when central concepts are difficult to capture using keyword searches [50]. Forward searching was carried out in Scopus on July 26, 2022, and included the following skill use measures: the Behavioral Activation for Depression Scale [51] and its short form [52], Cognitive Behavioral Therapy Skills Questionnaire [53], Skills of Cognitive Therapy [54], Competencies of Cognitive Therapy Scale [55], Frequency of Actions and Thoughts Scale [56], and Ways of Responding Scale [57].

### Inclusion and Exclusion Criteria

Inclusion and exclusion criteria were developed using the participants, intervention, comparator, outcome, and study design framework [58]. Only peer-reviewed articles published in English between 2000 and 2022 were included in this review.

#### Participants

Studies targeting adolescent or adult samples with a mean age of ≥12 years were eligible for inclusion. Articles were excluded if the mean age of the sample was <12 years or if the study primarily targeted people with a physical health condition. Primary studies were not required to screen participants for the presence of elevated anxiety or depressive symptoms to be eligible for inclusion given evidence that prevention programs can result in symptom improvements among people who do not have clinical levels of depression or anxiety [59,60].

#### Intervention

Articles were eligible for inclusion if they tested a stand-alone CBT intervention delivered via a digital platform that was designed to reduce or prevent symptoms of depression or anxiety. Interventions were classified as CBT if they were described as such by the study authors and included cognitive restructuring as a core component. Interventions could be delivered with or without guidance. Articles were excluded if the intervention (1) was not CBT (eg, behavioral activation, acceptance and commitment therapy, or cognitive behavioral stress management), (2) did not include depression or anxiety as the main intervention target (eg, a program that primarily focused on chronic pain but also included a depression outcome), (3) used technology but was delivered at a clinic or in the laboratory (ie, not a distal intervention), (4) was delivered as part of stepped care or as adjunctive therapy, or (5) was primarily delivered by a health professional in person or via videoconference or email (eg, the main intervention was in person, but an SMS text message component was included).

#### Comparator

Eligible interventions could be compared with an active (eg, other intervention or attention placebo) or inactive (eg, waitlist or no-intervention) control group. Uncontrolled studies were excluded.

#### Outcomes of Interest

Articles were included if they reported a quantitative measure of skill enactment or knowledge acquisition. The selection of eligible measures for this review was based on definitions provided within existing engagement frameworks [33,34]. In terms of skill enactment, articles were eligible for inclusion if they (1) included a measure designed to assess the implementation of treatment strategies and techniques (eg, self-reported frequency of practicing cognitive and behavioral skills) or (2) captured data on the practice of techniques and strategies within the intervention program (eg, thought record completion or tracking of exposures conducted in reality). We excluded general measures of coping, emotion regulation, and self-efficacy as these measures do not specifically target CBT skills and may include concepts and strategies not targeted for change in CBT [35]. We also excluded papers that only reported indicators of active engagement (eg, the number of program activities, exercises, or tools used) as these measures do not provide direct information on skill enactment.

Regarding knowledge acquisition, articles were eligible for inclusion if they reported a measure of (1) actual learning via a knowledge test or (2) self-reported learning. For this review, measures of general mental health literacy (ie, measures targeting awareness of the causes, epidemiology, symptoms, diagnosis, and treatment of mental disorders [61]) were included in addition to measures explicitly aimed at assessing knowledge of CBT principles, as CBT programs typically include psychoeducational content aimed at increasing knowledge and understanding. Indicators of program satisfaction or acceptability (eg, how understandable an intervention was) and treatment self-efficacy (eg, confidence or perceived ability to learn intervention content) were excluded.

#### Study Design

Primary and secondary reports of RCTs were eligible for inclusion. Cluster and factorial designs were eligible for inclusion, as were pilot and feasibility trials. Non-RCTs and observational studies (eg, cross-sectional, cohort, and case-control designs) were excluded.

### Study Selection

The search results were uploaded to EndNote (version 20; Clarivate Analytics), and duplicate records were removed automatically and through hand searching. Study selection was completed in 3 stages. At stage 1, titles and abstracts were screened by 1 of 2 reviewers (HJ or AT) and discussed with the last author (LF) to exclude irrelevant records. A third reviewer (GF) screened 10% of the abstracts to confirm that the review criteria had been applied consistently (percentage of agreement=99.6%; Cohen κ=0.97). At stage 2, full-text articles were uploaded to the Covidence systematic review software (Veritas Health Innovation) and coded by the first author (HJ) according to whether the studies (1) evaluated a CBT-based program and (2) reported a measure of skill enactment or knowledge acquisition (“yes,” “no,” or “unclear”). Relevant construct definitions and inclusion criteria were piloted with 50 full-text articles and refined through discussion within the review team before completion of this stage. A second reviewer screened 10% of the full-text articles to check for coder bias at stage 2, which did not result in the inclusion of any additional manuscripts (percentage of agreement=98.4%; Cohen κ=0.90). At stage 3, all articles coded as “yes” or “unclear” were referred to a third stage for double screening by 2 reviewers (HJ and GF) against all inclusion criteria. Discrepancies were resolved through discussion, and a third author (LF) was consulted if an agreement could not be reached.

### Data Extraction and Coding

A data extraction form was developed in Microsoft Excel (Microsoft Corp) and pilot-tested on 5 papers. Data extraction was completed by the first author (HJ), and accuracy was confirmed by a second author (GF). The key data elements extracted included skill enactment and knowledge acquisition data to address the objectives of the review (described in the *Strategy for Data Synthesis* section). In addition, we extracted the following descriptive data: country where the study was conducted, study design and type of comparison, participant characteristics and recruitment setting, intervention details and the type of technology, the length of the treatment period and intervention structure, the presence of guidance, and adherence or use data. We only extracted information for eligible interventions, and data for all eligible interventions were extracted if more than one eligible intervention was evaluated in the study. We accessed and cited primary reports or protocol papers to retrieve information if relevant data were not available in the paper under review.

The presence of guidance was categorized as guided (support related to treatment content was provided), unguided (no support related to treatment content provided), or supported (support was provided by educational staff for interventions delivered in educational settings, but this support was not related to treatment content). We included a *supported* category as interventions delivered in school settings involve a captive audience. Studies were also categorized according to the age group of the sample. Age classifications were established based on the mean age of the trial sample and included adolescents (aged 12-17 years), young adults (aged 18-24 years), and adults (aged ≥25 years). These classifications are consistent with the age categorizations used by the Australian Institute of Health and Welfare [62] and the legal age in Australia.

### Risk of Bias

The methodological quality of the included studies was independently assessed by 2 reviewers (HJ and LF) using the Joanna Briggs Institute Critical Appraisal Checklist for RCTs [63]. The checklist includes 13 items to evaluate randomization procedures, allocation concealment, selection bias, blinding, postassignment attrition, outcome measurement, and appropriateness of the statistical analyses and trial design. Items were scored as “Yes,” “No,” “Unclear,” or “Not applicable” according to the guidelines provided by Tufanaru et al [63]. For secondary reports, we referred to primary outcome papers to obtain ratings where necessary. Disagreements between reviewers were resolved through discussion until a consensus was reached. A score of 1 was awarded for each item adequately addressed in the paper, with possible scores ranging from 0 to 13.

### Strategy for Data Synthesis

The studies were not pooled for meta-analysis because of methodological diversity related to the measurement tools used to evaluate skill enactment and knowledge acquisition, as well as the use of different statistical approaches to determine the associations among skill enactment, knowledge acquisition, and mental health outcomes. Instead, descriptive narrative synthesis was conducted to address the review questions per our PROSPERO registration (CRD42021275270). All eligible measures of skill enactment and knowledge acquisition were recorded and tabulated, with tables ordered by symptoms or disorder targeted. In addition, we recorded any data on change in skill enactment or knowledge acquisition outcomes at postintervention measurement or follow-up (eg, between- or within-group comparisons or assessments of self-reported skill practice or learning), any analysis of the association between skill enactment or knowledge acquisition and primary or secondary mental health outcomes (eg, via correlation, regression, or mediation analyses), and any investigation of potential predictors of skill enactment or knowledge acquisition (eg, via regression or between-group analyses).

Definitions provided in the literature on CBT skill use [35], knowledge [64,65], and DMHI engagement [24] and information provided by the study authors were used to classify and summarize methods of measuring skill enactment and knowledge acquisition (review question 1). These classifications were used to group studies to address the subsequent review questions. Descriptive information regarding whether the study reported a significant between- or within-group effect for skill enactment or knowledge acquisition at postintervention measurement was reported to examine the effectiveness of digital CBT programs in improving skill enactment and knowledge acquisition (review question 2). Between-group analyses comparing intervention and control conditions within studies were prioritized in reporting as they provide greater certainty regarding the relationship between intervention exposure and subsequent skill enactment or knowledge change. Where possible, effect sizes using the pretest n value were calculated as the mean difference between treatment and control groups at postintervention measurement or follow-up divided by the pooled SD of the baseline scores. All estimates were adjusted using a Hedges *g* correction [66]. Consistent with Luo et al [67], imputed data values were used if both complete case and imputed values were provided. Negative effect sizes indicated that the comparison group performed better than the treatment group on skill enactment or knowledge acquisition.

Significant and nonsignificant findings were also summarized to examine the association between mental health outcomes and skill enactment or knowledge acquisition (review question 3) and investigate predictors of skill enactment or knowledge acquisition (review question 4). Predictor variables could include participant characteristics (eg, age and sex), disease-specific effects (eg, baseline symptom levels), intervention characteristics (eg, presence of guidance), and components of intervention engagement (eg, module completion). Previous systematic reviews of predictors of adherence or engagement [68] were used to establish broad categories of predictors. For each synthesis, all indicators of skill enactment or knowledge acquisition were summarized if multiple indicators were reported in the study. For studies reporting both intention-to-treat (ITT) and completer analyses, only ITT results were reported. In addition, if studies reported results for subscale scores in addition to total scores on skill enactment or knowledge acquisition measures, we only reported total scores to aid interpretation of the results, although changes in subscales are noted in Multimedia Appendix 3 [36,61,64,69-114]. Relevant results for follow-up time points are also summarized in Multimedia Appendix 3.

## Results

### Study Selection Process

**Figure 1:**
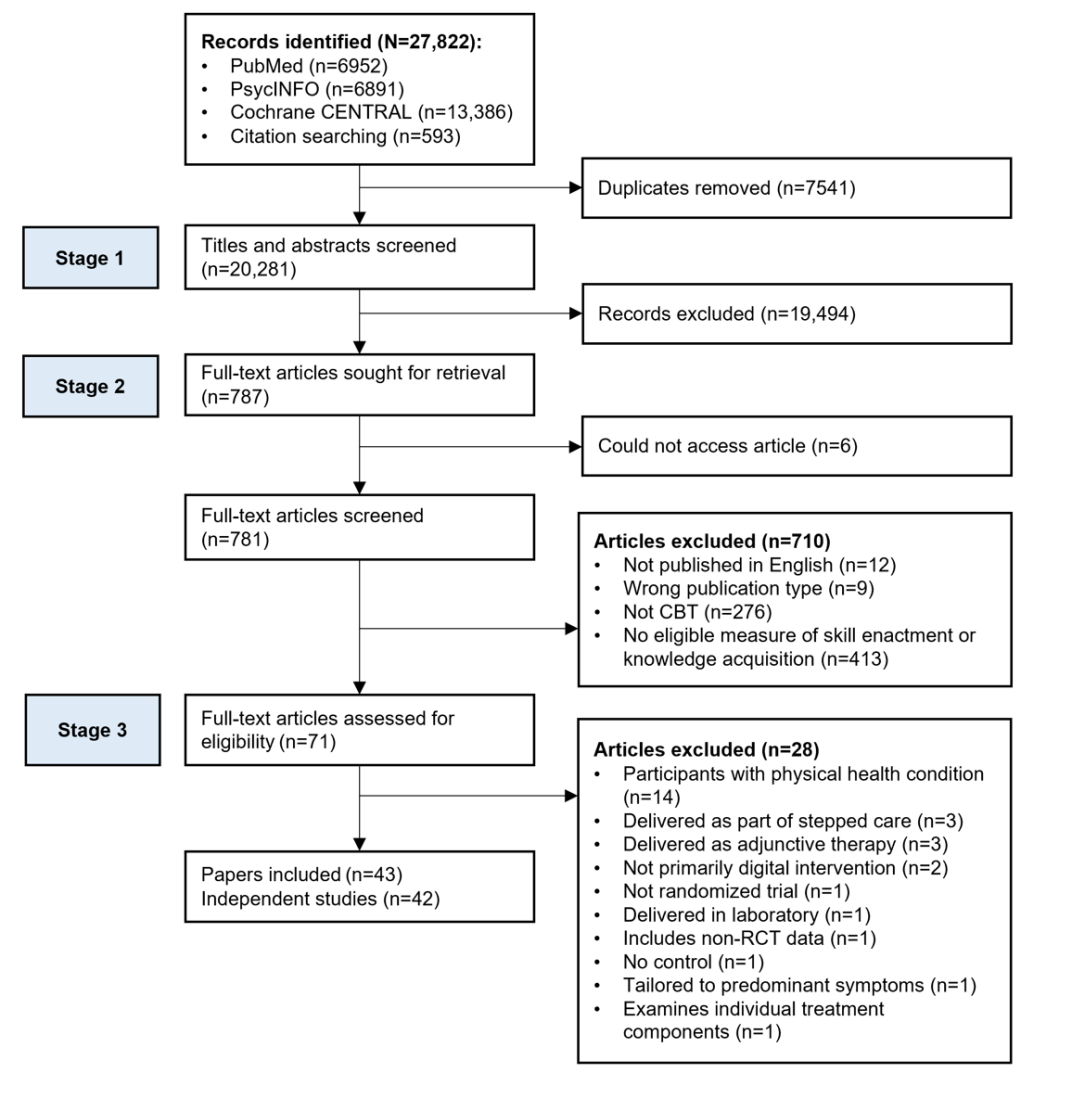
Flow diagram of study screening and inclusion. CBT: cognitive behavioral therapy; RCT: randomized controlled trial.

A total of 27,822 records were retrieved from the database and forward citation searches. After removing duplicates, 20,281 titles and abstracts were screened. Full texts were retrieved for 781 articles, and 43 (5.5%) papers [36,61,64,69-108] were included in the review (Figure 1). A study that appeared to meet the inclusion criteria was excluded as the trial was designed to test individual treatment components rather than an entire program of treatment, resulting in a 32-condition design [115]. On 2 occasions, more than one paper was published from a single trial, and both provided data on our outcomes of interest [89,90,101,102]. These manuscripts are reported together to describe study characteristics. In addition, one of these papers included 2 distinct trials [101]. These trials are reported separately. Therefore, 42 independent studies (reported in 43 papers) were included in the review.

### Study Characteristics

Study characteristics and outcomes of the review are reported in Multimedia Appendix 3. The studies were conducted in Europe (16/42, 38%), Australia (13/42, 31%), the United States (10/42, 24%), and Asia (3/42, 7%). Most studies (38/42, 90%) were parallel RCTs, although 5% (2/42) were cluster RCTs, 2% (1/42) were factorial design studies, and 2% (1/42) were described as intervention studies. Control groups were classified as waitlist (14/42, 33%), usual care (5/42, 12%), attention control (4/42, 10%), or enhanced usual care (2/42, 5%). In 21% (9/42) of the studies, participants were assigned to a waitlist control group but also received access to usual care, attention control materials, enhanced usual care, or a web-based discussion group. A total of 19% (8/42) of the studies compared ≥2 active treatments only.

### Sample Characteristics

A total of 10,078 participants were included in the 42 studies (ie, the number analyzed). Among the 41 studies that reported the number of participants by group assignment, 5881 participants received a stand-alone digital CBT intervention. The sample sizes ranged from 43 to 1236 (mean 239.95, SD 278.91; median 149.50). Most studies (30/42, 71%) recruited participants from the community, although 14% (6/42) recruited from educational settings, 10% (4/42) recruited from health care settings, and 5% (2/42) recruited from occupational settings. Studies (36/42, 86%) were typically conducted with adults, although 10% (4/42) included adolescents, and 5% (2/42) included young adults. Among the 31 studies where the mean age of the total sample was reported, the mean participant age ranged from 15.00 to 51.60 years (mean 31.90, SD 8.21 years). The proportion of female participants in the study samples ranged from 16.1% to 100% (median 76.59%), and 17% (7/42) of the studies included only female or pregnant participants, all of which focused on maternal or perinatal mental health.

### Intervention Characteristics

#### Symptoms or Disorders Targeted

Studies were categorized according to the symptoms or disorders targeted, as specified by the study authors. The most common conditions or symptoms targeted were depression (14/42, 33%), social anxiety (9/42, 21%), or depression and anxiety (5/42, 12%). Other symptoms or disorders targeted included panic disorder and agoraphobia (3/42, 7%), postnatal depression (3/42, 7%), perinatal anxiety and depression (2/42, 5%), and anxiety (1/42, 2%). A total of 12% (5/42) of the interventions targeted multiple mental health concerns. Almost half (19/42, 45%) of the trials focused on symptom reduction (ie, the study screened for and targeted individuals with elevated symptom levels not yet meeting diagnostic criteria), 38% (16/42) focused on treatment (ie, the study screened for and targeted individuals meeting diagnostic criteria for a depressive or anxiety disorder), and 17% (7/42) focused on prevention (ie, the study did not screen participants for elevated symptom levels). A total of 57 eligible interventions were tested in the 42 studies.

#### Type of Technology

Most studies (33/42, 79%) evaluated internet-based interventions, with access primarily provided via a computer. The remaining studies tested smartphone apps (3/42, 7%), internet-based programs with an adjunct smartphone app (2/42, 5%), a computerized intervention (delivered via CD-ROM; 1/42, 2%), a conversational agent (1/42, 2%), a multiplatform intervention (1/42, 2%), and an internet-based intervention delivered via a smartphone app versus a computer (1/42, 2%).

#### Presence of Guidance

A nearly equal number of studies evaluated only guided (19/42, 45%) or unguided (17/42, 40%) interventions, and 5% (2/42) of the studies tested supported interventions (self-guided interventions delivered in a supported environment such as a classroom). In addition, 5% (2/42) of the studies compared digital CBT delivered with and without guidance, 2% (1/42) compared digital CBT delivered with different types of guidance, and another study (1/42, 2%) tested unguided digital CBT compared with 2 types of guidance.

#### Intervention Length

Intervention length ranged from 2 weeks to 4 months (mean 8.21, SD 3.78; median 8.00). Among the 34 studies that specified the number of modules or sessions, the number of core modules ranged from 3 to 17 (mean 7.21, SD 2.46; median 7.50).

### Methodological Quality of the Included Studies

The number of studies that satisfied each of the quality items is shown in Table 1, and full information is provided in Multimedia Appendix 4 [36,61,64,69-108,116]. Overall, the quality of the included studies was good, with most studies (36/42, 86%) meeting ≥8 criteria (mean 8.81, SD 1.31). Most studies described adequate randomization (31/42, 74%) and allocation concealment procedures (33/42, 79%). The treatment groups were typically similar at baseline, or the subsequent analyses clearly accounted for any observed differences. Blinding was the primary issue in the included studies, with no study meeting the blinding of participants or blinding of outcome assessors criteria as all included participant-reported scales. In addition, only approximately one-third (15/42, 36%) of the studies met the blinding of those delivering treatment criterion as they evaluated unguided interventions. All studies (42/42, 100%) treated compared groups identically and measured outcomes in the same way across the groups. Most studies (33/42, 79%) reported incomplete follow-up, although patterns of loss to follow-up were generally adequately described and analyzed, and most studies (35/42, 83%) used ITT analyses. Despite all studies using validated outcome measures, close to half (22/42, 52%) did not provide clear information to determine whether outcomes were measured reliably in the study. Most studies (39/42, 93%) described appropriate statistical procedures, and the trial design was considered appropriate in all studies, with 2/2 (100%) of cluster RCTs using analytical techniques to account for clustering in the analyses.

**Table 1:** Number (and percentage) of the included studies meeting the criteria on the Joanna Briggs Institute (JBI) Critical Appraisal Checklist for Randomized Controlled Trials (n=42).

Item number	JBI checklist item	Studies, n (%)
1	True randomization	31 (74)
2	Allocation concealment	33 (79)
3	Treatment groups similar at baseline^a^	36 (86)
4	Participants blind to treatment assignment	0 (0)
5	Those delivering the intervention blind to treatment assignment^b^	15 (36)
6	Outcome assessors blind to treatment assignment	0 (0)
7	Treatment groups treated identically	42 (100)
8	Follow-up complete or differences between groups adequately described and analyzed	33 (79)
9	Participants analyzed in the groups to which they were randomized	35 (83)
10	Outcomes measured in the same way for treatment groups	42 (100)
11	Outcomes measured reliably	22 (52)
12	Appropriate statistical analysis	39 (93)
13	Appropriate trial design and any deviations accounted for	42 (100)

^a^In 26% (11/42) of the studies, observed differences between treatment groups were accounted for in the analyses.

^b^Studies testing unguided programs wherein treatment was delivered entirely on the web met this criterion by default.

### Studies Reporting a Measure of Skill Enactment or Knowledge Acquisition

Of the 43 papers, 28 (65%) reported ≥1 measure of skill enactment [36,74,77-82,84,85,88,90,92-106,108], fourteen (33%) reported ≥1 measure of knowledge acquisition [61,64,69-73,75,83,86,87,89,91,107], and 1 (2%) reported a measure of both skill enactment and knowledge acquisition [76].

Of the 29 studies reporting a measure of skill enactment, 26 (90%) were conducted with adults, 2 (7%) were conducted with adolescents, and 1 (3%) was conducted with young adults. The studies evaluated guided (15/29, 52%), unguided (10/29, 34%), or supported (2/29, 7%) interventions, and 7% (2/29) compared ≥1 guided and unguided intervention.

Of the 15 studies reporting a measure of knowledge acquisition, most (n=11, 73%) were conducted with adults, although 3 (20%) included adolescent samples, and 1 (7%) included a young adult sample. The studies evaluated guided (5/15, 33%), unguided (8/15, 53%), or supported (1/15, 7%) interventions, and 7% (1/15) compared guided and unguided interventions.

### Review Question 1: What Methods Have Been Used to Measure Skill Enactment and Knowledge Acquisition?

#### Skill Enactment

The methods used to measure skill enactment varied across the studies. These included formal skill enactment measures captured via log data (13/29, 45%) and standardized (8/29, 28%) or intervention-specific (9/29, 31%) questionnaire measures. Among the studies reporting an indicator of formal skill enactment, most targeted social anxiety (7/13, 54%) or panic disorder and agoraphobia (2/13, 15%). Of the studies reporting a standardized or intervention-specific questionnaire, studies typically evaluated interventions for depression (7/17, 41%), perinatal mental health (4/17, 24%), or depression and anxiety (2/17, 12%). Regardless of the measure used, skill enactment was assessed as frequency (eg, how often a person performed skills over the past week or the number of in-program activities completed) or time spent practicing skills in all studies except 1 (28/29, 97%), which also assessed quality of skill enactment. See Table 2 for a breakdown of the specific ways in which skill enactment was measured in the included studies.

**Table 2:** Summary of methods used to investigate skill enactment in the included studies (n=29).

Type of skill enactment measure and measure of skill enactment			Times reported^a^, n (%)
**Formal skill enactment**			
	Tracked exposures	7 (24)	
	Cognitive restructuring exercises	6 (21)	
	Anxiety diaries	4 (14)	
	Activity planning	2 (7)	
	Behavioral experiments	2 (7)	
	Attentional training exercises	1 (3)	
	Relaxation exercises	2 (7)	
	Global indicators	4 (14)	
**Standardized questionnaire^b^**			
	Behavioral activation skills	6 (21)	
	Cognitive skills	1 (3)	
	Cognitive and behavioral skills	2 (7)	
**Intervention-specific questionnaire^b^**			
	Time spent practicing skills (single item)	5 (17)	
	Frequency of practicing skills (single item)	2 (7)	
	Frequency of practicing specific skills (multi-item)	3 (10)	
	Successful use of skills^c^	1 (3)	

^a^Numbers do not add up to 29 as some studies include more than one indicator of skill enactment.

^b^All standardized and intervention-specific questionnaires relied on participant self-reports unless otherwise stated.

^c^On the basis of coach reports.

#### Knowledge Acquisition

Methods used to measure knowledge acquisition included objective tests (9/15, 60%) designed to measure declarative knowledge about CBT principles or mental health literacy, as well as questionnaire measures (6/15, 40%) to assess perceived learning or knowledge. Studies that reported a measure of knowledge acquisition typically targeted symptoms of depression (8/15, 53%) or anxiety and depression (2/15, 13%). Table 3 provides a breakdown of the ways in which knowledge acquisition was measured in the included studies.

**Table 3:** Summary of methods used to investigate knowledge acquisition in the included studies (n=15).

Type of knowledge acquisition measure and measure of knowledge acquisition^a^			Times reported^b^, n (%)
**CBT^c^ knowledge test**			
	Multiple-choice items^d^	3 (20)	
	True-or-false items	2 (13)	
	Multiple-choice + true-or-false items^d^	1 (7)	
**Mental health literacy test**			
	Multiple-choice items	1 (7)	
	True-or-false items	1 (7)	
	Helpfulness ratings	1 (7)	
	Multiple-choice items + helpfulness ratings	1 (7)	
	True-or-false items + vignettes	1 (7)	
**Questionnaire measure**			
	Perceived learning (single item)	4 (27)	
	Perceived knowledge of specific treatment components (multi-item)	2 (13)	

^a^Knowledge tests and questionnaire measures were completed by the participants in all studies.

^b^Numbers do not add up to 15 as some studies include more than one measure of knowledge acquisition.

^c^CBT: cognitive behavioral therapy.

^d^In addition to the total number of correct answers, 50% (3/6) of the studies reporting CBT knowledge tests reported weighted scores based on the level of certainty associated with a given response.

### Review Question 2: Are Digital CBT Programs Effective in Improving Skill Enactment and Knowledge Acquisition?

#### Skill Enactment

A total of 8 studies evaluated skill enactment from baseline to postintervention measurement, all of which used standardized questionnaire measures to assess the frequency of enacting skills. Of these 8 studies, 6 (75%) reported significant findings in favor of the intervention group at postintervention measurement [36,76,84,94,101], whereas 2 (25%) did not [95,98]. Significant findings were reported for 4 (80%) out of 5 studies that evaluated interventions for symptoms of depression, 1 (50%) out of 2 studies that evaluated interventions for postnatal depression, and 1 study that evaluated an intervention for symptoms of anxiety and depression. The Hedges *g* ranged from 0.09 to 2.08 (median 0.76). Effect sizes could not be calculated for 1 study because of insufficient data regarding the direction of the effect [95].

#### Knowledge Acquisition

A total of 10 studies evaluated knowledge acquisition from baseline to postintervention measurement. The results are grouped by type of knowledge measure.

##### CBT Knowledge

A total of 6 studies on 10 eligible interventions examined whether CBT knowledge improved at postintervention measurement, and all (6/6, 100%) reported significant improvements across all eligible interventions using between-group [61,64,69,73,76] or within-group analyses [72]. The studies evaluated interventions targeting symptoms of depression (n=4), social anxiety (n=1), or anxiety and depression (n=1). For studies with available data (4/6, 67%), the Hedges *g* ranged from 0.17 to 0.74 (median 0.67). Effect sizes could not be calculated for 2 studies because of insufficient data or a lack of a control condition [64,72].

##### Mental Health Literacy

A total of 5 studies on 7 eligible interventions examined whether mental health literacy improved at postintervention measurement. Of these 5 studies, 2 (40%) reported significant findings in favor of the intervention group at postintervention measurement across all eligible interventions [75,91], 2 (40%) reported mixed findings (where significant findings were associated with some but not all interventions or measures of literacy) [61,64], and 1 (20%) reported no significant findings [70]. Significant or mixed findings were reported for 3 studies that evaluated interventions for symptoms of depression and 1 study for stress, depression, anxiety, and substance abuse. Nonsignificant findings were reported in 1 study, which targeted anxiety, depression, and well-being. The Hedges *g* ranged from −0.17 to 0.92 (median 0.07). Effect sizes could not be calculated for 1 study because of insufficient data [64].

##### Self-reported Knowledge Acquisition

A total of 2 studies examined whether self-reported knowledge improved at postintervention measurement, with 1 (50%) reporting significant findings in favor of the intervention group across all learning areas [86], whereas the other (n=1, 50%) found no significant change [87]. Both studies evaluated interventions for symptoms of depression. The Hedges *g* ranged from −0.03 to 0.49 (median 0.32).

### Review Question 3: Is There an Association Between Mental Health Outcomes and Skill Enactment or Knowledge Acquisition?

#### Skill Enactment

A total of 12 studies evaluated the relationship between skill enactment and postintervention mental health outcomes, of which most (n=7, 58%) examined indicators of formal skill enactment and all but 2 (17%) were conducted with adult samples. The results in the following sections are grouped by type of skill enactment measure.

##### Standardized Questionnaire Measures

Four studies examined whether frequency of skill enactment mediated the effect of the intervention on postintervention mental health outcomes using standardized questionnaire measures. Of these 4 studies, 3 (75%) found that skill enactment significantly mediated improvements in mental health outcomes at postintervention measurement [36,101], and 1 (25%) found that frequency of enacting cognitive but not behavioral activation skills mediated subsequent change in mental health outcomes [84]. All 4 studies investigated reductions in depressive symptoms, with 1 study also investigating improvements in anxiety and life satisfaction.

##### Formal Skill Enactment Measures

A total of 7 studies on 11 eligible interventions investigated the association between indicators of formal skill enactment and mental health outcomes at postintervention measurement. As some studies included multiple analyses (eg, multiple skill enactment measures investigated or >1 eligible intervention evaluated), results are organized around the specific measure used to predict mental health outcomes and summarized in terms of the number of analyses. We focus on indicators showing significant results in ≥2 analyses, although Table 4 summarizes all analyses.

**Table 4:** Summary of formal skill enactment indicators used to predict postintervention mental health outcomes (n=7).

Formal skill enactment indicator	Studies^a^, n/N (%)	Positive analyses^b^, n/N (%)
Tracked exposures	3/3 (100)	4/5 (80)
Global indicator	2/3 (67)	2/5 (40)
Cognitive restructuring exercises^c^	1/4 (25)	1/6 (17)
Anxiety diaries	1/2 (50)	1/4 (25)
Activity planning	0/1 (0)	0/1 (0)
Relaxation tools	0/2 (0)	0/3 (0)

^a^Number of studies reporting ≥1 significant positive associations between a formal skill enactment indicator and mental health outcomes over the total number of studies investigating that indicator.

^b^Number of analyses reporting a significant positive association between a formal skill enactment indicator and mental health outcomes over the total number of analyses.

^c^One analysis showed a negative association between cognitive restructuring exercises and improvement in stress at postintervention measurement, although this indicator was associated with significant improvements in anxiety and stress at follow-up.

Overall, at least one positive finding was reported in 71% (5/7) of the studies. However, only the number of exposure exercises completed (4/5, 80% of analyses in 3/3, 100% of the studies) and global indicators (2/5, 40% of analyses in 2/3, 67% of the studies) were positively associated with improvement in mental health outcomes at postintervention measurement in ≥2 analyses. At least 1 significant positive finding was reported in each of the 4 studies evaluating improvements in social anxiety symptoms [77,79,100,105] and in 1 study evaluating improvements in depression, anxiety, and stress [85]. Nonsignificant or negative findings were reported in studies evaluating improvements in depression [108] or anxiety, depression, and stress [96]. A total of 1 study provided insufficient detail to determine the exact number of significant analyses [74] and was excluded from the synthesis.

##### Intervention-Specific Questionnaire

A total of 1 study examined whether frequency of enacting intervention-specific skills was associated with improvement in mental health outcomes at postintervention measurement. Results were largely nonsignificant; skill enactment was associated with improvements in support-seeking coping but not in depression, anxiety, well-being, or emotion regulation [90].

#### Knowledge Acquisition

A total of 4 studies evaluated the association between knowledge acquisition and mental health outcomes at postintervention measurement. The results in the following sections are grouped by type of knowledge measure.

##### CBT Knowledge

A total of 3 studies on 6 eligible interventions examined whether CBT knowledge acquisition was associated with improvement in mental health outcomes at postintervention measurement. None of the studies reported significant findings for the primary outcomes of depression (n=2), anxiety (n=1), or social anxiety (n=1) [69,72,73], although 1 study reported a small significant correlation between knowledge acquisition and improvement in the secondary outcome of social anxiety [69].

##### Mental Health Literacy

A total of 1 study on 2 eligible interventions investigated whether improvements in mental health literacy were associated with the primary outcomes of depression, anxiety, and mental well-being at postintervention measurement, with no significant findings reported [70].

### Review Question 4: What Predictors of Skill Enactment and Knowledge Acquisition Have Been Identified?

#### Skill Enactment

##### Overview

In total, 13 studies investigated potential predictors of frequency or time spent using skills, of which 6 (46%) examined formal skill enactment measures, 4 (31%) examined intervention-specific questionnaires, and 3 (23%) examined standardized questionnaire measures. The results in the following sections are grouped according to type of predictor and focus on significant findings. As with review question 3, the results are reported in terms of the number of studies and analyses.

##### Intervention Content

In total, 2/13 (15%) studies examined whether factors related to intervention content were associated with time spent using skills (ie, CBT with or without exposure and CBT with or without mindfulness), and the results were not significant [88,99].

##### Intervention Features

A total of 2/13 (15%) studies on 4 eligible interventions examined whether the presence of specific intervention features was associated with frequency of enacting skills, and there were mixed results. The delivery of an adjunct skills-based app during an internet-based intervention (compared with sequential delivery of the same app; 1/1, 100% of the analyses) [77] and the absence of a group news feed and accountability features (compared with the presence of these features; 1/2, 50% of the analyses) [81] were associated with increased skill enactment.

##### Therapeutic Approach

A total of 2/13 (15%) studies examined whether the therapeutic approach was associated with frequency or time spent using skills (ie, cognitive restructuring vs self-compassion intervention and internet-based CBT vs internet-based exposure therapy), and the results were largely nonsignificant (1/5, 20% of the analyses) [103,104].

##### Presence and Type of Guidance

In total, 2/13 (15%) studies on 5 eligible interventions investigated whether the presence and type of guidance were associated with skill enactment frequency. Individual therapist guidance, compared with group-based guidance, was found to be positively associated with skill enactment (2/2, 100% of the analyses) [100], but the presence or option of guidance was not [74].

##### Psychological Factors

A total of 2/13 (15%) studies examined whether psychological factors were associated with frequency of enacting skills, with both studies finding that improvements in skill enactment were associated with improvements in negative thinking (2/2, 100% of the analyses) and savoring (2/2, 100% of the analyses) [101].

##### Baseline Symptoms

A total of 2/13 (15%) studies investigated whether baseline symptoms predicted the frequency of skill enactment, with neither study reporting significant findings [90,96].

##### Program Use

A total of 1/13 (8%) studies examined whether completion of a fixed number of modules was associated with skill enactment frequency [98], and the results were not significant.

#### Knowledge Acquisition

A total of 2 studies examined predictors of knowledge acquisition. The results are grouped according to the type of predictor.

##### Learning Support

In total, 1/2 (50%) studies examined whether learning support integrated into a web-based program predicted CBT knowledge acquisition, and the results were significant (1/1, 100% of the analyses) [72].

##### Presence of Guidance

A total of 1/2 (50%) studies investigated whether weekly therapist support was associated with improvements in CBT knowledge, and the results were not significant [72].

##### Group Assignment

A total of 1/2 (50%) studies evaluated whether assignment to the active intervention condition was associated with higher levels of perceived learning relative to an attention control program, and the results were significant (1/1, 100% of the analyses) [71].

## Discussion

### Principal Findings

This review aimed to systematically examine the literature on skill enactment and knowledge acquisition in the context of digital CBT interventions for symptoms of depression or anxiety. In total, 43 papers (reporting on 42 independent trials) were included, of which 29 (67%) reported a measure of skill enactment and 15 (35%) reported a measure of knowledge acquisition.

#### Methods Used to Measure Skill Enactment

Most of the research on engagement with digital CBT interventions for depression and anxiety has not measured skill enactment. Despite the use of broad inclusion criteria to identify skill enactment measures, only approximately 6.4% (29/456) of eligible papers reported a quantitative measure of skill enactment and were included in this review. In contrast, a previous review of adherence to manualized web-based interventions found that 85% of primary publications included information on program use [24]. Eligible measures of skill enactment were also difficult to identify in many cases, either because the measures were insufficiently described in the study methods and results or because they were not reported under engagement or adherence headings. Nevertheless, we identified 3 broad approaches to measuring skill enactment: formal skill enactment indicators, standardized questionnaire measures, and intervention-specific questionnaires. Formal skill enactment measures were used more frequently than standardized or intervention-specific questionnaire measures. Furthermore, the reporting of each of these measures appeared to vary depending on the symptoms or disorders targeted. More than half (7/13, 54%) of the studies that examined formal skill enactment measures evaluated interventions for symptoms of social anxiety disorder, whereas most studies (13/17, 76%) that reported standardized or intervention-specific questionnaires targeted symptoms of depression, perinatal mental health, or depression and anxiety.

This review identified some weaknesses in current methods of measuring skill enactment. Only standardized questionnaires of behavioral activation skills, single items measuring “global” skill enactment, and adherence measures derived from exposure diaries or cognitive restructuring exercises were examined in ≥5 studies. As digital CBT programs target a range of adaptive skills, the use of unidimensional measures may not sufficiently capture the multifaceted nature of these interventions or enable examination of the differential impact of enacting specific skills. A related issue is the relatively common use of automatically captured log data (ie, formal skill enactment measures) as a proxy for skill enactment (although we do not suggest that this was the intention of the study authors). Although these measures provide helpful information on the frequency of using specific intervention tools, they cannot capture the various ways in which people can engage with skills outside their interaction with a program. For example, people might schedule pleasant events or achievement activities on their phones or personal calendars rather than using the tools provided in the program itself. Therefore, it is important to implement measures that reflect the breadth of recommended skills and the various ways in which people can enact them.

The studies also varied in the methods used to report skill enactment data (eg, the number of formal exercises completed and percentage of participants able to regularly perform exercises), and nearly all studies (28/29, 97%) reported the frequency or amount of time spent practicing skills based on retrospective self-reports. These approaches overlook key aspects of skill enactment outlined in the literature on CBT and emotion regulation [35,37], including the competence with which skills are enacted by participants (ie, skill enactment quality) and the number or range of skills used (ie, skill enactment quantity), and they may be susceptible to social desirability and memory biases that hinder accurate recall or reporting. It will be important for studies to examine skill enactment quality and quantity, and in the case of guided interventions, it may be possible for therapists or other supporters to observe and rate the quality of skill enactment indirectly via evaluations of thought records, web-based practice sessions, or other homework materials. Hundt et al [35] provide a review of existing therapist-rated measures of skill enactment quality used in face-to-face CBT. The literature would also benefit from ecological momentary assessment methods that capture skill enactment as it occurs in real time to overcome reliance on retrospective reporting and improve measurement precision. Together, these methods may help address several important research questions, such as “what type of skill enactment is necessary for outcomes” (eg, frequent use of skills, use of a broad range of skills, or competent use of skills) and “which skills are most important for change?”

#### Changes in Skill Enactment and Association With Mental Health Outcomes

The number of studies that examined changes in skill enactment (n=8) or effects on mental health outcomes (n=12) was small and, again, limited to assessments of skill enactment frequency using questionnaire measures or log data. Nevertheless, some promising findings were reported. Most studies that evaluated changes in skill enactment (6/8, 75%) found that levels of skill enactment increased at postintervention measurement among participants exposed to interventions relative to the comparison groups. Estimated effect sizes were generally medium to large. Overall, these data suggest that interventions targeting depression are effective in improving the frequency of skill enactment. Interventions for postnatal depression or anxiety and depression combined may also be effective, but the findings were mixed [94,95] or limited to only 1 study [36]. One or more significant positive findings were also reported in 83% (10/12) of the studies that examined the relationship between skill enactment and mental health outcomes at postintervention measurement. In particular, each of the studies (4/4, 100%) reporting standardized measures provided evidence for an association between intervention exposure, greater frequency of enacting skills, and postintervention improvement in mental health outcomes [36,84,102], although the outcomes were almost exclusively limited to improvement in depressive symptoms. Overall, these findings are generally consistent with engagement models that propose that participation in an intervention will produce improvements in skills and outcomes [32-34], as well as with some cognitive behavioral theories on the mechanisms of action in CBT [35].

In contrast, studies that addressed mental health outcomes in relation to formal skill enactment measures provided mixed evidence. Only the number of tracked exposures and global skill enactment indicators were positively associated with outcomes in ≥2 analyses, whereas other indicators (eg, cognitive restructuring and activity planning) were not found to be consistently related to outcomes or were addressed in only 1 analysis. This result is perhaps not surprising given the similarly mixed findings for comparable program use measures, such as number of diary entries, tools used, or activities [22]*,* and further highlights the importance of adopting a comprehensive approach to measuring skill enactment that enables examination of what participants do offline*.* Intervention-specific questionnaires can provide a thorough assessment of skill enactment and may be particularly well suited to evaluating programs that target a range of therapeutic techniques and strategies when appropriate standardized measures are not available, but their utility in predicting mental health outcomes has not yet been elucidated, with only 1 pilot study exploring the association between intervention-specific skill enactment and mental health outcomes [90]. Future studies that use intervention-specific measures should explicitly examine change in skill enactment and its association with treatment outcome.

#### Predictors of Skill Enactment

Similar to the selection of skill enactment measures, there was considerable variation in the predictors and types of skill enactment studied. The same predictor was only investigated in more than one study on 2 occasions. There was some evidence that participants used skills more often if they demonstrated improvements in negative thinking and savoring [101]. However, similar to some previous reviews on predictors of adherence [17,68], mental health symptoms at baseline were not found to predict skill enactment [90,96]. Other factors considered in 1 study each included program use [98] and various intervention-related factors such as comparisons between therapeutic approaches and the presence and type of support [74,77,81,88,99,100,103,104]. Given the small number of studies that considered individual predictors of skill enactment, the findings described in this review could be related to the specific study samples or methods. Research has also neglected to examine theoretically relevant predictors of skill enactment. Some level of knowledge acquisition is likely to be a prerequisite for enacting skills, but this still needs to be investigated. In addition, there may be a range of factors that influence the likelihood of enacting skills once users have acquired knowledge. For example, the theory of planned behavior states that behavior change depends not only on an individual’s ability to carry out a behavior but also on their readiness or motivation to do so, which in turn depends on attitudes, social influences, and self-efficacy [117]. Future studies that use a theory-driven approach are warranted to clarify the preliminary relationships described in this review and examine the influence of underlying motivational variables.

#### Methods Used to Measure Knowledge Acquisition

Only 3.3% (15/456) of eligible articles reported a quantitative measure of knowledge acquisition. Among these studies, objective tests (eg, multiple-choice or true-or-false tests and objectively scored helpfulness ratings) designed to measure declarative knowledge about CBT or mental health literacy comprised almost two-thirds (9/15, 60%) of the measures. Self-report questionnaires on perceived learning were also reported, although these measures were typically limited to single items. Regardless of the type of measure reported, more than half (8/15, 53%) of the included studies evaluated interventions for depression. Some limitations of existing approaches to measuring knowledge acquisition include the fact that most studies (9/15, 60%) only considered declarative knowledge (ie, knowledge of facts and information) measured using recognition-based tasks (eg, multiple-choice questions). Other forms of learning and knowledge may also be important to consider, such as more implicit and procedural knowledge (ie, knowledge of how to perform a skill), as well as the ability to generalize and apply learning to novel situations [118,119]. Possible ways to capture these constructs include the use of open-ended items to measure free recall performance, reviews of homework, hypothetical scenarios, and vignette designs [40,73,120].

#### Changes in Knowledge Acquisition and Association With Mental Health Outcomes

Most studies (8/10, 80%) that evaluated changes in knowledge found that intervention group participants significantly improved their levels of knowledge at postintervention measurement. The studies that found at least one positive effect included all the studies (6/6, 100%) that used a test of CBT knowledge, 80% (4/5) of the studies evaluating mental health literacy, and 50% (1/2) of the studies evaluating perceived knowledge acquisition. Overall, these findings indicate that participants can learn and recognize novel information about mood and anxiety disorders and underlying therapeutic principles in digital CBT. These interventions may also be effective in improving perceived knowledge and learning, but the findings were mixed [86,87]. As would be expected given the focus of the included interventions on CBT principles and information, effect sizes were generally the largest for measures of CBT-specific knowledge. However, even for these measures, the magnitude of change was generally modest (ie, intervention group participants answered, on average, only 1 to 2 more items correctly than at baseline [61,69,73]), perhaps because of relatively high levels of preexisting knowledge.

There was no evidence that knowledge acquisition, assessed using tests of CBT knowledge or mental health literacy, was associated with improved mental health outcomes at postintervention measurement. Thus, participants can learn the treatment content, but they do not necessarily benefit in terms of symptom reduction. Overall, this pattern of results is in line with studies evaluating digital CBT interventions targeting other common mental disorders such as eating disorders [121,122], as well as with suggestions that knowledge acquisition alone may not be sufficient to improve symptoms [34]. Instead, it is the active application of skills (ie, skill enactment), which may depend not only on knowledge change but also on an individual’s motivation to implement mental health actions, that has been suggested to be important for mental health outcomes [34,73], an idea that is largely supported by the results of this review. However, we advise caution in interpreting the findings in this way. Some research from other areas has shown that knowledge acquisition can predict symptom improvements in certain instances, for example, for individuals with low levels of preexisting CBT knowledge and in cases where changes in knowledge confidence, not accuracy, are correlated with outcomes [122]. Furthermore, some of the included studies demonstrated that improvements in both CBT knowledge and mental health literacy can be maintained at the 6- to 12-month follow-ups [61,72]. It is possible that these enduring improvements facilitate ongoing recovery from current episodes of depression and anxiety or confer a protective effect against future episodes via improvements in an individual’s ability to recognize symptoms within themselves or others and respond to challenges or by facilitating timely help seeking.

#### Predictors of Knowledge Acquisition

Only 2 studies on knowledge acquisition examined potential predictors of knowledge acquisition. Significant predictors included the use of learning support strategies and assignment to the active intervention rather than the attention control group, whereas therapist guidance was nonsignificant [71,72]. However, the small number of studies that considered predictors of knowledge acquisition makes it challenging to draw conclusions about the reliability of these findings, and further research is warranted. The potential role of learning support may be especially important to consider as Berg et al [72] found that building pedagogical techniques into program modules led to small improvements not only in CBT knowledge but also in mental health outcomes despite a nonsignificant relationship between these outcomes. These findings suggest that developing a better understanding of how best to support people in learning treatment strategies and techniques may help improve outcomes. Harvey et al [38] provide an overview of cognitive support strategies that may enhance memory for psychological treatments.

### Research Gaps and Future Directions

This review highlighted several gaps in the current state of knowledge in this area. Overall, there was a lack of research that examined skill enactment and knowledge acquisition in the context of digital CBT. Very few studies (6/42, 14%) reported on skill enactment and knowledge acquisition among adolescents and young adults, and most studies (33/42, 79%) evaluated internet-based interventions accessed via a computer. Similar to other authors [24,25,73], we encourage researchers to measure and report skill enactment and knowledge acquisition in future studies of digital CBT and examine outcomes and predictors related to these constructs.

However, future research must go beyond simply measuring skill enactment and knowledge acquisition to also address measurement issues. A key limitation of the literature identified by this review is the heterogeneity in how knowledge acquisition, and especially skill enactment, was measured and analyzed. Heterogeneity was evident not only in the specific construct examined (eg, skill enactment frequency vs quality and CBT knowledge vs mental health literacy) but also in the mode of assessment and the way in which data were reported and analyzed. This heterogeneity hinders the pooling of data for quantitative synthesis and may be due in part to a lack of specific theoretical guidance or standards available to inform the selection, analysis, and reporting of skill enactment and knowledge acquisition measures in DMHIs. To facilitate consistency going forward, we recommend that definitions of skill enactment and knowledge acquisition in DMHIs and the subsequent selection of measures are theory-driven. For example, the literature on CBT skill use and emotion regulation provides clear conceptual definitions of skill enactment and its distinct components (eg, frequency, quantity, and quality) that can inform the selection of appropriate measures [35,37]. A theory-driven approach will be aided by developing robust and broadly applicable scales to measure skill enactment and knowledge acquisition in DMHIs more consistently and considering how these scales are related to outcomes. In the context of skill enactment, recent work by Titov et al [123] and Bisby et al [124] to develop and test a measure of daily actions associated with psychological health may align well with conceptual definitions of skill enactment frequency. In addition, psychometric research comparing different methods of measuring skill enactment and knowledge acquisition is needed to examine whether each is measuring a unitary construct or whether there are distinct types of skill enactment and knowledge acquisition beyond those highlighted within conceptual frameworks.

In addition, although the overall quality of the included studies was good, some methodological issues were evident, with most or all studies not meeting quality assessment criteria related to blinding of participants (0/42, 0%), outcome assessors (0/42, 0%), or those delivering treatment (15/42, 36%). This is an inherent problem with RCTs of psychological interventions [125]. However, it is possible to implement blinding in these interventions in some cases (eg, by not informing participants of which treatment is expected to be most effective in studies including active treatment or attention control comparisons) [125]. It is also notable that more than half (22/42, 52%) of the studies did not provide sufficient information to determine whether outcomes were measured reliably (eg, reporting the Cronbach α for the collected data) despite many reporting estimates from external sources. Unreliable measurement of outcomes can weaken the validity of inferences made about statistical relationships [63]. It is essential that studies explain in sufficient detail the methods used to allow for accurate assessments of study quality.

### Limitations of This Review

This review has some limitations. The inclusion of an English-language restriction and single screening of titles, abstracts, and initial full texts may have increased the risk of selection bias. Although double screening of 10% of the records at each stage did not result in the inclusion of any additional papers, it is possible that some publications were excluded erroneously. It is also possible that some studies were excluded because of insufficient or unclear reporting of the study methods or results. The review was further limited to RCTs of stand-alone digital CBT interventions for depression and anxiety. Thus, our findings cannot be generalized to open-access settings, other mental health problems, stepped-care interventions, or psychotherapeutic treatments other than CBT. Nevertheless, the examination of knowledge acquisition and skill enactment is likely to be important in any skills training intervention, and this review can inform the development of future studies in this area. In addition, we could not conduct a quantitative synthesis of skill enactment and knowledge acquisition because of methodological diversity in the included studies, meaning that we could not estimate the average effect of skill enactment or knowledge acquisition on depression and anxiety outcomes, nor could we examine the factors that modify these effects. Future meta-analytic studies will be essential to understand therapeutic mechanisms in digital CBT and advance future interventions but will require greater methodological consistency.

### Conclusions

In digital interventions for mental health problems, it is essential to consider engagement as a concept that extends beyond program use to encompass actions that are implemented outside initial engagement with an intervention. Given that most studies on engagement with DMHIs focus on program use, this review addresses a substantial gap by focusing on skill enactment and knowledge acquisition during or following program use. This review demonstrated that digital CBT interventions for depression and anxiety appear to be effective in improving skill enactment frequency; levels of CBT knowledge; and, to a lesser extent, mental health literacy. However, only skill enactment frequency was found to be associated with positive mental health outcomes. Few studies have investigated predictors of skill enactment and knowledge acquisition, and those that have done so have mostly been limited to investigations of intervention-related factors and reported null results. This review calls for a more systematic and theory-based approach to studying the role of skill enactment and knowledge acquisition in DMHIs for depression and anxiety. The findings of this review can inform the development and selection of skill enactment and knowledge acquisition measures and promote the inclusion of these types of measures in future studies evaluating DMHIs.

## Appendix

Multimedia Appendix 1 PRISMA (Preferred Reporting Items for Systematic Reviews and Meta-Analyses) checklist.

Multimedia Appendix 2 Search strategies for PubMed, PsycINFO, and Cochrane CENTRAL.

Multimedia Appendix 3 Results of the review.

Multimedia Appendix 4 Quality assessment.

## Abbreviations

CBT: cognitive behavioral therapy
DMHI: digital mental health intervention
ITT: intention-to-treat
PRISMA: Preferred Reporting Items for Systematic Reviews and Meta-Analyses
RCT: randomized controlled trial
