# Supplementary material for: Skill Enactment and Knowledge Acquisition in Digital Cognitive Behavioral Therapy for Depression and Anxiety: Systematic Review of Randomized Controlled Trials
Source: J Med Internet Res. 2023 May 31;25:e44673. doi: 10.2196/44673 (PMC10267790; doi:10.2196/44673)
Supplement: Multimedia Appendix 2 [file jmir_v25i1e44673_app2.pdf]

Full Search Strategy 2000 to 2022

	**Ovid PsycINFO** 1806 to July Week 2 2022<br>Search date: 26 July 2022	
#	**Searches**	**Results**
1	(internet or web or online or icBT or i-CBT or eHealth or e-Health or cCBT or c-CBT or digital or computer or mHealth or m-Health or mobile or smartphone or virtual).ti,ab.	281941
2	(internet or smartphone or computers).mp.	89765
3	1 or 2	294352
4	(depression or depressive or depressed or mood disorder or mood disorders or mood disordered or affective disorder or affective disorders or affective disordered or mood or affective or dysthymic or dysthymia).ti,ab.	444460
5	(depression or mood disorder or affect or dysthymic disorder).mp.	583699
6	4 or 5	693001
7	(anxiety or anxious or phobic or phobia or phobias or panic or obsess* or compulsi* or worry).ti,ab.	270885
8	(anxiety or phobic disorders or panic).mp.	278768
9	7 or 8	320140
10	6 or 9	851470
11	3 and 10	47379
12	treatment effectiveness evaluation.sh.	26868
13	(random* and trial*).tw.	80381
14	(random* and allocate*).tw.	5997
15	double blind.tw.	24382
16	single blind.tw.	2316
17	12 or 13 or 14 or 15 or 16	118952
18	clinical trial.id.	2445
19	clinical trial*.tw.	39868
20	((singl* or doubl* or trebl* or tripl*) adj5 blind).tw.	26528
21	(clin* adj25 trial*).ti,ab.	49172
22	placebo*.tw.	43247
23	placebo*.id.	6029
24	placebo*.ti,ab.	42749
25	wait list*.ab.	2848
26	random*.ti,ab.	226331
27	18 or 19 or 20 or 21 or 22 or 23 or 24 or 25 or 26	278488
28	17 or 27	295251
29	11 and 28	7657
30	limit 29 to (human and yr="2000 -Current")	6891
	**Cochrane CENTRAL**<br>Search date: 26 July 2022	
#	**Searches**	**Results**
#1	("internet" OR "web" OR "online" OR "iCBT" OR "i-CBT" OR "eHealth" OR "e-Health" OR "cCBT" OR "c-CBT" OR "digital" OR "computer" OR	89742

	"mHealth" OR "m-Health" OR "mobile" OR "smartphone" OR "virtual"):ti,ab	
#2	([mh internet] OR [mh smartphone] OR [mh computers]):ti,ab	6736
#3	#1 OR #2	90708
#4	("depression" OR "depressive" OR "depressed" OR "mood disorder" OR "mood disorders" OR "mood disordered" OR "affective disorder" OR "affective disorders" OR "affective disordered" OR "mood" OR "affective" OR "dysthymic" OR "dysthymia"):ti,ab	102329
#5	([mh depression] OR [mh "mood disorders"] OR [mh affect] OR [mh "dysthymic disorder"]):ti,ab	27959
#6	#4 OR #5	105562
#7	("anxiety" OR "anxious" OR "phobic" OR "phobia" OR "phobias" OR "panic" OR "obsess*" OR "compulsi*" OR "worry" OR "agoraphobia"):ti,ab	58407
#8	([mh anxiety] OR [mh "phobic disorders"] OR [mh panic]):ti,ab	10655
#9	#7 OR #8	60023
#10	#6 OR #9	133083
#11	#10 AND #3	13801
#12	#10 AND #3<br>with Publication Year from 2000 to 2022, in Trials	13531
	**PubMed**<br>Search date: 26 July 2022	
#	**Searches**	**Results**
#1	"internet"[Title/Abstract] OR "web"[Title/Abstract] OR "online"[Title/Abstract] OR "iCBT"[Title/Abstract] OR "i-CBT"[Title/Abstract] OR "eHealth"[Title/Abstract] OR "e-Health"[Title/Abstract] OR "cCBT"[Title/Abstract] OR "c-CBT"[Title/Abstract] OR "digital"[Title/Abstract] OR "computer"[Title/Abstract] OR "mHealth"[Title/Abstract] OR "m-Health"[Title/Abstract] OR "mobile"[Title/Abstract] OR "smartphone"[Title/Abstract] OR "virtual"[Title/Abstract]	942,049
#2	"internet"[MeSH Terms] OR "smartphone"[MeSH Terms] OR "computers"[MeSH Terms]	174,970
#3	#1 OR #2	1,015,870
#4	"depression"[Title/Abstract] OR "depressive"[Title/Abstract] OR "depressed"[Title/Abstract] OR "mood disorder"[Title/Abstract] OR "mood disorders"[Title/Abstract] OR "mood disordered"[Title/Abstract] OR "affective disorder"[Title/Abstract] OR "affective disorders"[Title/Abstract] OR "affective disordered"[Title/Abstract] OR "mood"[Title/Abstract] OR "affective"[Title/Abstract] OR "dysthymic"[Title/Abstract] OR "dysthymia"[Title/Abstract]	594,241
#5	"mood disorders"[MeSH Terms] OR "depression"[MeSH Terms] OR "mood disorders"[MeSH Terms] OR "affect"[MeSH Terms] OR "dysthymic disorder"[MeSH Terms]	287,619
#6	#4 OR #5	650,451
#7	"anxiety"[Title/Abstract] OR "anxious"[Title/Abstract] OR "phobic"[Title/Abstract] OR "phobia"[Title/Abstract] OR "phobias"[Title/Abstract] OR "panic"[Title/Abstract] OR "obsess*"[Title/Abstract] OR "compulsi*"[Title/Abstract] OR "worry"[Title/Abstract] OR "agoraphobia"[Title/Abstract]	290,202
#8	"anxiety"[MeSH Terms] OR "phobic disorders"[MeSH Terms] OR "panic"[MeSH Terms]	116,131

#9	#7 OR #8	316,546
#10	#6 OR #9	813,632
#11	#10 AND #3	42,037
#12	"randomised controlled trial"[Title/Abstract] OR "randomized controlled trial"[Title/Abstract] OR "randomised control trial"[Title/Abstract] OR "randomized control trial"[Title/Abstract] OR "rct"[Title/Abstract] OR "random trial"[Title/Abstract] OR "randomised trial"[Title/Abstract] OR "randomized trial"[Title/Abstract] OR "randomised"[Title/Abstract] OR "randomized"[Title/Abstract] OR "controlled trial"[Title/Abstract] OR "control trial"[Title/Abstract]	755,053
#13	#11 AND #12	7,023
#14	#13 AND ("2000/01/01"[Date - Publication] : "3000"[Date - Publication])	6,952
	**Scopus**<br>**Forward Citation Searches (published CBT skills usage measures)**<br>Search date: 26 July 2022	
	**Measure/ Citation**	**Results**
	Behavioral Activation for Depression Scale [46]	269
	Behavioral Activation for Depression Scale-Short Form [47]	112
	Cognitive-Behavioral Therapy Skills Questionnaire [48]	47
	Skills of Cognitive Therapy [49]	55
	Competencies of Cognitive Therapy Scale [50]	42
	Frequency of Actions and Thoughts Scale [51]	10
	Ways or Responding Scale [52]	58
